# Effect of inhaled hydrosoluble curcumin on inflammatory markers in broncho-alveolar lavage fluid of horses with LPS-induced lung neutrophilia

**DOI:** 10.1186/s40248-015-0010-7

**Published:** 2015-04-15

**Authors:** Charlotte Sandersen, Dorothee Bienzle, Simona Cerri, Thierry Franck, Sandrine Derochette, Philippe Neven, Ange Mouytis-Mickalad, Didier Serteyn

**Affiliations:** Equine Clinic, Department of Clinical Sciences, Faculty of Veterinary Medicine, University of Liège, Liège, Belgium; Department of Pathobiology, Ontario Veterinary College, University of Guelph, Guelph, ON N1G 2W1 Canada; Center for Oxygen Research and Development, University of Liege, Sart Tilman, B6a, 4000 Belgium

**Keywords:** Airway inflammation, Large animal model, Lipopolysaccharide, Curcumin, NDS27

## Abstract

**Background:**

Horses commonly suffer from chronic respiratory disease and are also used in large animal models of spontaneous or induced airway inflammation. The anti-inflammatory properties of curcumin are largely described but its low bioavailability precludes its clinical use. NDS27, a lysin salt of curcumin incorporated in beta-cyclodextrine, has high bioavailability and can be administered by inhalation. The aim of this study was to investigate the effects of inhaled NDS27 on inflammatory cytokines and proteins in the broncho-alveolar lavage fluid using a model of neutrophilic airway inflammation.

**Methods:**

Airway neutrophilia was induced in eight horses by inhalation of lipopolysaccharides (LPS). Horses were treated with either inhalation of NDS27 or with placebo in a randomized cross-over design. Broncho-alveolar lavages were performed 6 hours after stimulation with LPS. Percentage of neutrophils, concentrations of IL-1β, TNF-α, IL-6, Club cell secretory protein, myeloperoxidase (MPO) and elastase (ELT) concentrations were determined.

**Results:**

LPS stimulation induced significant increases in neutrophil counts and concentrations of IL-6 (70.2 ± 66.0 pg/ml), TNF-α (43.9 ± 31.2 pg/ml), MPO (580.9 ± 327.0 ng/ml) and ELT (27.6 ± 16.7 ng/ml). Treatment with NDS27 significantly prevented the increase in active and total MPO (216.4 ± 118.1 ng/ml) and ELT (5.9 ± 3.2 ng/ml) while there was a trend towards reduced IL-6 concentration.

**Conclusions:**

Results show that, although not reducing neutrophil recruitment, NDS27 largely abolishes LPS-induced neutrophil degranulation. Reduced levels of ELT and MPO, as well as reduced MPO activity may have beneficial effects via a reduced production of reactive oxygen species implicated in chronic inflammation and airway remodeling.

## Background

Horses commonly suffer from recurrent airway obstruction (RAO) or heaves, which is a severe, potentially debilitating, chronic inflammatory airway disease typically affecting middle-aged horses [1]. From a clinical point of view, the disease is characterized by periods of acute exacerbation that are interspersed by periods of remission when horses are kept away from the causative environment [1]. Initiated following exposure to organic dusts, molds, and lipopolysaccharides (LPS) in hay [2], acute exacerbations are characterized by neutrophilic airway inflammation, coughing, periods of labored breathing at rest and exercise intolerance due to bronchospasm and mucus accumulation in the airways. The exact immunological processes responsible for the airway hyper-reactivity and the persistent airway inflammation are still under investigation [3,4] and several studies have focused on cytokine expression profiles [5-9]. It seems that the equilibrium between pro-and anti-inflammatory factors is disturbed and shifted towards a pro-inflammatory profile. Club cell secretory protein (CCSP) is a recognised inhibitor of inflammation in the lung. Both, the gene expression and protein secretion are depleted in the airways of symptomatic RAO horses compared with control [10]. This finding may reflect the entry of CCSP into luminal neutrophils, potentially inhibiting oxidative burst and enhancing phagocytosis [11]. Activated neutrophils release oxidative enzymes such as NADPH oxidase, myeloperoxidase (MPO) and NO synthase that are responsible for the *in vivo* generation of reactive nitrogen and oxygen reactive species (RNOS) and several other proteolytic enzymes such as collagenase, cathepsin and elastase (ELT) [12,13]. Activation of inflammatory cells by exposure to exogenous stimulants plays a major role to tip the balance towards an excess of RNOS production [14,15].

Recently, the levels of RNOS in RAO-affected and clinically healthy horses have been investigated and the therapeutic use of agents that may reduce the oxidative stress has been suggested [13]. One of these potential agents is curcumin, a natural phenolic compound, which has largely documented anti-inflammatory properties (for review see Prasad et al. [16]). It has been shown that curcumin has the ability to reduce the production of RNOS by stimulated neutrophils through acting as an RNOS scavenger but also as a direct inhibitor of MPO and NADPH oxidase [17,18]. A highly water-soluble form of curcumin called NDS27 was developed by our team (WO 2009144220 A1). NDS27 is a combination of a salt of curcuminoid derivative and hydroxypropyl-beta-cyclodextrin. As curcumin, NDS27 has the ability to inhibit the oxidant response of neutrophils and the activity of MPO [19].

NDS27 has been investigated in RAO-affected horses [20]. Inhalation with NDS27 resulted in decreased neutrophil percentages and reduced myeloperoxidase concentration in broncho-alveolar lavage fluids (BALF) relative to sham-treated horses.

LPS-induced neutrophilic inflammation plays a key role in the pathogenesis of RAO in horses [2] and lung inflammation in other species. Experimental inhalation of LPS is commonly used as a model of neutrophilic lung inflammation mimicking natural disease. Inhaled endotoxins are an important cause of human pulmonary disease, with the severity of pulmonary inflammation and clinical symptoms experienced by subjects exposed to organic dusts being related to the endotoxin concentration in the inhaled dust [21]. Consistent with endotoxin inhalation studies in humans and other species [21], inhalation of 20, 200 and 2000 μg soluble *Salmonella typhimurium* Ra60 LPS induced a dose-dependent airway neutrophilia, with BALF neutrophil numbers increasing approximately 50-fold in heaves horses at 6 h after the high dose challenge [22].

The horses’ susceptibility to develop airway inflammation, as well as the ease by which the airway inflammation can be induced in healthy horses, make the horse an ideal model for testing drugs by inhalation. Although not very popular compared to murine models of respiratory disease, horses have some considerable advantages over smaller laboratory animals [23]. Horses naturally develop heaves which is characterized by stable dust-induced inflammation, bronchospasm, and remodelling. Horses can be evaluated during well-controlled natural antigen exposure allowing the study of disease mechanisms in the asymptomatic and symptomatic stages. Further, the disease can be followed over longer periods to evaluate the cumulative effect of repeated episodes of clinical exacerbation and of long-term treatment. Horses can easily be exposed to inhalation challenges and repeated broncho-alveolar lavages can be performed in the same animal. LPS challenge and especially naturally occurring heaves share similarities with the human asthma and the equine model is currently being used to evaluate airway remodelling [23].

The aim of this study was to test the effect of NDS27 on the BALF concentration of several pro-inflammatory cytokines, the neutrophil enzymes MPO and elastase, and the anti-inflammatory protein CCSP in an equine model of LPS-induced lung neutrophilia.

## Methods

### Animals

The study was approved by the institutional committee of animal use (Commission d’Ethique Animale de l’Université de Liège, Protocol 11-1137). Eight clinically healthy adult Standardbred horses with no history of RAO were included in this study. All horses were maintained on pasture for at least two months without supplementation of hay and underwent a thorough physical examination, complete blood count and airway endoscopy before the beginning of the study.

### Study protocol

The control BALs were performed on eight horses without any treatment while they were kept on pasture (CTRL). Three weeks later, the horses were randomly assigned to both stimulation and placebo (STIM) or both stimulation and treatment (NDS27) groups. After a washout period of three weeks horses received the other treatment.

Horses of both groups were challenged with 2 mg of LPS (Sigma-Aldrich, Belgium) diluted in 4 ml of NaCl (0.9%) given via inhalation (SaHoMa, Nebu-tec, Germany) at T0. They received placebo or treatment at one hour before and two and four hours after challenge. Each dose of NDS27 contained 4 mg of curcumin, 49.8 mg of hydroxy-propylbeta-cyclodextrin and 1.1 mg of lysine base. Each dose of the placebo treatment contained 49.8 mg of hydroxy-propylbeta-cyclodextrin and 1.1 mg of lysine base. Each dose was diluted in 4 ml of NaCl 0.9% before administration by inhalation (Flexineb, Nortex, UK). In all horses, BALs were performed 6 hours after stimulation (T + 6 h).

### Broncho-alveolar lavages

All BALs were performed as follows: horses were sedated with detomidine (Domidine, Eurovet, Heusden-Zolder, Belgium) at a dose of 10 μg/kg intravenously. A broncho-alveolar catheter (BAL catheter 240 cm, Surgivet, Smith Medical, Zaventem, Belgium) was introduced naso-tracheally and forwarded until it became locked in a bronchus. Warmed sterile saline solution (240 ml) was injected and gently aspirated. The amount of recovered fluid was recorded. From the recovered fluid, 9 ml were transferred to an EDTA-tube and centrifuged. The supernatant was kept frozen at-20°C until analyses. A different aliquot served for determination of total nucleated cell count with the help of a Thomas chamber. From the original BAL sample 100 μL were used to generate a centrifuged slide (Cytospin, Terumo, Belgium) that was Giemsa-stained. Two investigators unaware of the treatment performed differential cell counts by identifying 400 cells under light microscopy. The mean of the two counts were used for statistical analyses.

### Sandwich ELISA for equine TNF-α

BALF TNF-α concentration was determined by a sandwich ELISA kit designed and validated specifically for equine samples (Equine TNF-α, Genorise, USA). The assay was performed according to manufacturer’s instructions. Each sample was assayed twice and the mean value was calculated. Calibration samples and non-diluted EDTA samples were incubated 1 hour at 22°C.

### Sandwich ELISA for equine IL1-β and IL-6

BALF concentrations of IL1-β and IL-6 were determined by a sandwich ELISA kit designed and validated specifically for equine samples (Genorise, USA). The assay was performed according to manufacturer’s instructions. Each sample was assayed twice and the mean value was calculated. Calibration curves and non-diluted EDTA samples were incubated 2 h at 37°C.

### CCSP assay

Concentration of CCSP in BALF fluid was determined as described previously [10]. Briefly, 100 μL of reCCSP (0, 12.5, 25, 50, 100 and 200 ng/mL) or BALF were placed in wells overnight at 4°C. Next day, wells were washed, 100 μL of eCCSP-AB (a rabbit anti-equine CCSP antibody, diluted 1:350) was added for 1 hour at room temperature, wells were washed again, and horseradish peroxidase-labeled swine anti-rabbit polyclonal antibody (Dako) was added. Finally, 100 μL of tetramethylbenzidine (Pierce, Rockford, IL) was added to each well, plates were incubated for 15 minutes, and the reaction was terminated by addition of 100 μL of 0.5 M sulfuric acid. Absorbance was measured at 450 nm. Incubation of samples with pre-immune rabbit serum in lieu of eCCSP-AB served as a negative control, and readings from blank wells were used to determine background signal. All samples and standards were tested in triplicate. Standard curve analysis was used to extrapolate sample CCSP concentrations.

### Sandwich ELISA for equine myeloperoxidase (MPO)

Concentration of MPO in BALF was determined by a commercially available ELISA kit (Equine MPO ELISA kit, BiopTis, Belgium) developed by Franck at el., [24]. Equine MPO standards ranging from 0.78–50 ng/ml and EDTA BALF were added (100 μl) into the wells, and microplates were incubated overnight at 4°C. The secondary antibody was labeled with alkaline phosphatase and the revelation system used a paranitrophenyl phosphate-stabilized solution. The absorbance was directly proportional to the MPO captured by the primary antibody and, therefore, to the concentration of MPO in the sample. Each sample was assayed twice and the mean value was calculated.

### Sandwich ELISA for equine elastase (ELT)

A specific ELISA for equine neutrophil ELT developed by de la Rebière de Pouyade et al. (2010) [25] was used for measurement of ELT concentration in BALF (Equine ELT ELISA kit, BiopTis, Belgium). Equine elastase standards ranging from 1.875–60 ng/ml and EDTA BALF were added (100 μl) into the wells, and microplates were incubated overnight at 4°C. The secondary antibody was labeled with alkaline phosphatase and the detection system used a paranitrophenyl phosphate-stabilized solution. The absorbance was directly proportional to the ELT captured by the primary antibody and, therefore, to the concentration of ELT in the sample. Each sample was assayed twice and the mean value was calculated.

### Specific immuno extraction followed by enzymatic detection (SIEFED) assay for measurement of MPO activity

MPO activity was measured to determine the possible mechanism of action of NDS27. The SIEFED method allows the capture of MPO from biological fluids by microplate-coated specific antibodies. The fluid is eliminated by washings and the *in situ* activity of the enzyme is determined by a combination of a fluorogenic substrate (Amplex red) and a nitrite-based amplifier system. The primary antibody (rabbit anti-MPO IgG) was coated onto black microplate wells. Equine MPO standards (ranging from 0.25 to 6.4 mU/mL) and non-diluted samples containing MPO (100 μL) were added to the microplate and incubated for 2 hours at 37°C. After 3 washings, the peroxidase activity of MPO was detected by adding 100 μL of 40 μM Amplex red (10-acetyl-3, 7-dihydroxyphenoxazine), freshly prepared in phosphate buffer (50 mM) at pH 7.5 containing 10 μM H_2_O_2_ and 10 mM nitrite. Fluorescence was measured with a fluorescence plate reader (Fluoroscan Ascent, Fischer Scientific) at the excitation and emission wavelengths of 544 and 590 nm. Each sample was run in duplicate. The fluorescence value is directly proportional to the quantity of active MPO in the sample.

### Statistical analysis

An analysis of variance on repeated measurements was used for each parameter to compare the mean values of the control group, the STIM group and the NDS27 group. A sphericity test was used to assess the equality of variances of the differences between measurements [26]. The multiple pairwise comparisons were performed by a Bonferoni test. All the tests were performed with commercially available software (MedCalc Software, Oostende, Belgium) with the level of significant set at < 0.05.

## Results

**Figure 1:**
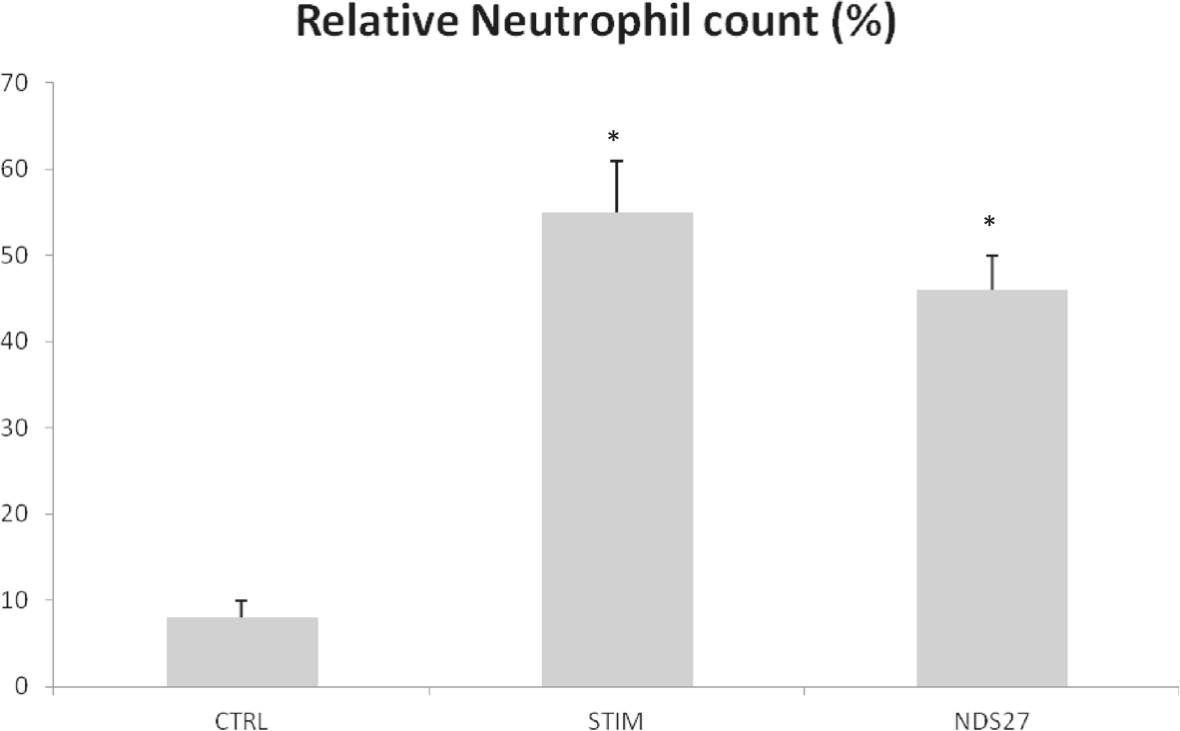
Relative neutrophil counts in broncho-alveolar lavage fluids from 8 horses at baseline, after stimulation with lipopolysaccharide aerosol and treatment with NDS27 or saline. CTRL, control group; STIM, stimulation with LPS and treatment with saline; NDS27, stimulation with LPS and treatment with NDS27. *Significantly different (p < 0.05) from CTRL group.

All horses tolerated challenge with LPS and inhalation of NDS27 without any sign of discomfort. Median total nucleated cell counts were 92.5 cells/μL (range: 75–115) for the control group, 325 cells/μL (range: 215–355) for the STIM group and 321 cells/μL (range: 285–360) for the NDS27 group. Relative neutrophil counts in BALF are displayed in Figure 1. Stimulation with LPS led to significantly higher mean relative neutrophil counts of 55 ± 6% in the stimulation only group and 46 ± 4% in the group treated with NDS27. Although lower in the NDS27 group, relative neutrophil count was not significantly different between the treatment groups.

**Figure 2:**
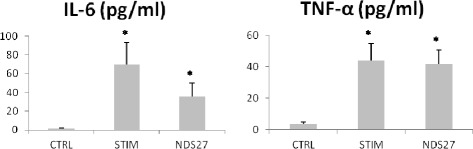
Concentrations of **(a)** interleukin-6 (IL-6) and **(b)** tumor necrosis factor-α (TNF-α) in broncho-alveolar lavage fluids from 8 horses at baseline, after stimulation with lipopolysaccharide aerosol and treatment with NDS27 or saline. CTRL, control group; STIM, stimulation with LPS and treatment with saline; NDS27, stimulation with LPS and treatment with NDS27. *Significantly different (p < 0.05) from CTRL group.

Mean concentrations (± standard error) of the cytokines of IL-6 and TNF-α are displayed in Figure 2a and b, respectively. Stimulation with LPS significantly increased concentrations of both cytokines in BALF. Although not statistically significant, there was a trend towards a decrease of IL-6 in the BALF of the NDS27 treatment group when compared to the STIM group (p = 0.06).

Half of the samples were below the limit of detection for the measurement of IL-1β. For the remaining samples, mean concentration of IL-1β was 6.09 ± 1.86 pg/ml in the CTRL group, while it was 0.75 ± 0.57 pg/mL in the STIM group and 2.76 ± 1.29 pg/mL in the NDS27 group. There were no significant differences between the three groups.

**Figure 3:**
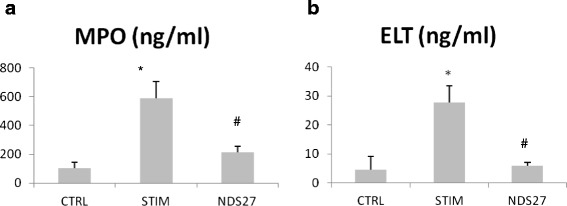
Concentration of **(a)** myeloperoxidase (MPO) and **(b)** elastase (ELT) in broncho-alveolar lavage fluids from 8 horses at baseline, after stimulation with lipopolysaccharide aerosol and treatment with NDS27 or saline. CTRL, control group; STIM, stimulation with LPS and treatment with saline; NDS27, stimulation with LPS and treatment with NDS27. *Significantly different (p < 0.05) from CTRL group. # Significant difference (p < 0.05) between the STIM and the NDS27 group.

**Figure 4:**
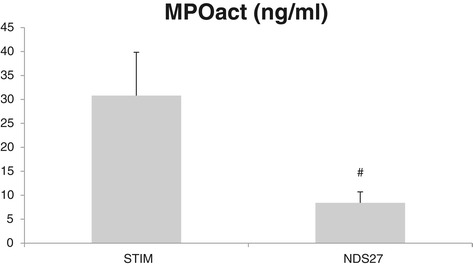
Concentration of active myeloperoxidase activity (MPOact) in broncho-alveolar lavage fluids from 8 horses after stimulation with lipopolysaccharide aerosol and treatment with NDS27 or saline. STIM, stimulation with LPS and treatment with saline; NDS27, stimulation with LPS and treatment with NDS27. # Significant difference (p < 0.05) between the STIM and the NDS27 group.

Results for mean concentration (± standard error) of MPO and ELT in BALF are displayed in Figure 3a and b, respectively. Both degranulation products are found in significantly higher concentrations in BALF of LPS-stimulated horses when compared to CTRL group. Treatment with NDS27 abolished this increase in led to significantly lower levels of both enzymes when compared to the placebo treated group. Mean concentrations of active MPO in BALF was significantly higher in the STIM group than in the NDS27 group, as displayed in Figure 4. Measurement of active MPO was unavailable in the CTRL group.

**Figure 5:**
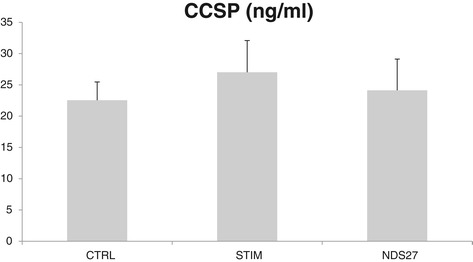
Concentration of Club cell secretory protein (CCSP) in broncho-alveolar lavage fluids from 8 horses after stimulation with lipopolysaccharide aerosol and treatment with NDS27 or saline. CTRL, control group; STIM, stimulation with LPS and treatment with saline; NDS27, stimulation with LPS and treatment with NDS27.

There were no significant differences between the three groups concerning concentration of CCSP in the BALF as shown in Figure 5.

## Discussion

Inhalation of LPS has previously been used to induce neutrophilic airway inflammation in humans [27], laboratory animals [28], and horses [22,29]. Several studies have shown that inhalation of LPS led to increased neutrophil counts and increased neutrophil ratios in BALF six hours after the challenge in healthy horses [22,29]. Concentration of TNF-α, IL-1, IL-6, IL-8, and IL-10 was increased in the BALF from horses challenged by LPS, although these changes were lower than in horses challenged with hay dust solution [22]. LPS inhalation increases the concentration of metallo-matrix proteinases in BALF suggesting their role in tissue remodeling [30]. Lipopolysaccharides are TLR4 agonists that activate NFκB dependent cytokine production [27] as well as induce release of TNF-α from macrophages. TNF-α is also responsible for neutrophil recruitment. In the present study, inhalation of LPS induced significant increases in the mean percentage of neutrophils, and in the mean concentrations of MPO, ELT, TNF-α and IL-6 in BALF when compared to control values. This finding is largely consistent with findings of earlier studies and underlies the usefulness of the universal model of LPS-induced neutrophilic airway inflammation. The neutrophilic enzymes MPO and ELT have not previously been studied in an LPS model in horses but MPO has been measured in BALF of horses naturally affected by RAO [31]; as well as in BALF of LPS-challenged rats [32] and mice [33]. In the present study no change in IL-1β was induced by stimulation with LPS aerosol. This is contradictory to previous studies in mice [34,35] but can be explained by insufficient sensitivity of the ELISA kit for detection of IL-1β in un-concentrated BALF. A potential effect of NDS27 on IL-1β could, therefore, not be investigated. Unfortunately, more sensitive methods such as real time polymerase chain reaction analysis (PCR) were not performed in the present study. PCR analysis would have also allowed investigation of other cytokines and degranulation markers such as matrix metalloproteinases.

We have previously showed that inhalation of NDS27 attenuates pulmonary neutrophilic inflammation in RAO-affected horses [20]. It was hypothesized that NDS27 mediated pro-apoptotic mechanisms inhibited the transcription factor NF-κB or decreased the neutrophil efflux into the lung. In the present work, the effect of inhaled NDS27 on inflammatory cytokines and enzymes in the BALF of horses submitted to a LPS-model of neutrophilic airway inflammation was evaluated. Treatment with NDS27 significantly reduced MPO, active MPO and ELT concentrations in BALF, compared to the non-treated group, while it had no significant effect on neutrophil recruitment. The previous study was performed on naturally affected RAO-horses, which are mainly suffering from signs of persistent airway inflammation. Horses had reduced relative neutrophil counts after one week of twice daily NDS27 treatment. Comparing the results of both studies, hay-dust induced RAO and LPS-induced lung neutrophilia, it seems that NDS27 is more effective in clearing persistent inflammation than impeding initial neutrophil recruitment.

Numerous studies showed that curcumins have the ability to control the inflammatory response via either the inhibition of the release or expression of inflammatory cytokines such Il-6, TNF- α, or by decreasing the activity and release of inflammatory enzymes. Indeed, *in vitro* studies showed that curcumin regulates activation of some receptors and transcription factors such as TLR4, activating protein-1 and NF-κB in stimulated monocytes and alveolar macrophages, thereby blocking expression of cytokine gene expression [36-38]. Down-regulation of intercellular signalling proteins, such as protein kinase C, may be another way in which curcumin inhibits cytokine production [37]. Additionally, the soluble form of curcumin abolished the stimulating effect of LPS on neutrophil stimulation and even decreased the release of two enzymes considered as typical markers of neutrophils stimulation such MPO and ELT. It was showed that the binding site for curcumin overlaps with the binding site for LPS and that curcumin binds at submicromolar affinity to the myeloid differentiation protein 2 (MD2), which is the LPS-binding component of the endotoxin surface receptor complex MD-2/TLR4 [39]. Since NDS27 could also enter the neutrophil [19], it is not excluded that the molecule could interfere with other intracellular pathways involved in the cascade of NADPH activation and degranulation. Beside a scavenging effect of curcumin and NDS27 on RNOS produced by neutrophils, our recent *in vitro* studies showed that curcumin had the ability to inhibit the assembly and activity of the neutrophil NADPH oxidase and that curcumin and its soluble form inhibited the release and the activity of MPO [18].

In the present study, treatment with NDS27 was initialised before stimulation with LPS27, which is not reflecting clinical conditions where insult would happen before the treatment. However, in experimental settings it is not uncommon to start treatment before the challenge with LPS. This has been done in a study, where curcumin was administered one hour before the administration of LPS in order to induce acute lung injury in mice [40]. Pre-treatment with curcumin reduced neutrophil recruitment, MPO activity, TNF-α concentration, and capillary leakage in response to LPS challenge when compared to treatment with placebo. Interestingly, treatment with curcumin performed almost as good as standard dexamethasone treatment. The rationale for investigating the effect on CCSP was based on its potent anti-inflammatory properties as demonstrated in several studies. Glucocorticoids are largely considered as mainstay therapy for equine RAO and are known to act on the CCSP promoter [41]. It has been shown previously that LPS decreases expression of CCSP in lung tissue and that this decrease is attenuated by pretreatment with plant-derived antioxidants such as polydatin, a natural precursor of resveratrol [42]. However, CCSP production is likely reduced in horses only as a result of long-standing lung inflammation and reduction in the number of Club cells [10]; therefore, absence of significant changes within a few hours after treatment was not unexpected.

## Conclusions

The main effect observed in this study is that NDS27 reduced neutrophil activation of LPS-induced airway inflammation, which subsequently reduced the oxidative and proteolytic enzyme concentrations in the BALF. Beneficial effects of a prolonged administration of NDS27 may be expected in clinical situations with persistent airway inflammation.

## Abbreviations

BAL: Broncho-alveolar lavage
BALF: Broncho-alveolar lavage fluid
CCSP: Club cell secretory protein
CTRL: Control group
eCCSP-AB: Equine club cell secretory protein–antibody
ELT: Elastase
LPS: Lipopolysaccharides
MPO: Myeloperoxidase
RAO: Recurrent airway obstruction
RNOS: Reactive nitrogen and oxygen species
STIM: Stimulation only group
